# COVID-19 Surveillance and Competition in Sport: Utilizing Sport Science to Protect Athletes and Staff during and after the Pandemic

**DOI:** 10.3390/jfmk5030069

**Published:** 2020-09-03

**Authors:** Joshua Hagen, Jason D. Stone, W. Guy Hornsby, Mark Stephenson, Robert Mangine, Michael Joseph, Scott Galster

**Affiliations:** 1 Rockefeller Neuroscience Institute, West Virginia University, Morgantown, WV 26505, USA; jason.stone1@hsc.wvu.edu (J.D.S.); scott.galster@hsc.wvu.edu (S.G.); 2 College of Physical Activity and Sport Science, West Virginia University, Morgantown, WV 26505, USA; william.hornsby@mail.wvu.edu; 3 Detroit Lions, Allen Park, MI 48101, USA; mark.stephenson@lions.nfl.net; 4 Athletic Department, University of Cincinnati, NovaCare Rehabilitation, Cincinnati, OH 45220, USA; manginre@ucmail.uc.edu; 5 Athletic Department, West Virginia University, Morgantown, WV 26505, USA; mike.joseph@mail.wvu.edu

**Keywords:** athlete monitoring, COVID-19, wellness, training load, wearables, digital health, sport science, recovery

## Abstract

The ongoing Coronavirus 2 (COVID-19) pandemic abruptly halted athletic competition and standard training practices, consequently generating great confusion surrounding when and how to safely reintroduce sports. Therefore, tangible solutions disseminated to performance staff, coaches, and athletes are warranted to ensure optimal levels of health and physical performance for all personnel during both the current social distancing standards as well as the impending return of competition despite continued risks. In this commentary, we offer strategies for utilizing technology and data tools as components of longitudinal COVID-19 surveillance based on ongoing research efforts as well as current guidance from governing bodies, while also serving the performance needs of the athletes and staff. Recommended data sources include digital symptom and well-being surveys, standardized and routine physical performance testing, sleep and sleep physiology monitoring, cognitive applications, and temperature. This system is flexible to numerous commercially available products and is designed for easy implementation that permits instant feedback provided directly to the athlete as well as their support staff for early intervention, ultimately mitigating COVID-19 risks. We will discuss multiple options, including examples of data, data visualizations and recommendations for data interpretation and communication.

## 1. Introduction

In March of 2020, the Coronavirus 2 (SARS-CoV-2 or COVID-19) global pandemic instantly halted normal daily living for virtually all of humanity and, more specifically, the sports world [1,2,3,4,5]. The impact of COVID-19 on athletics across the globe is profound considering the cessation of sports worldwide, and most notably, the cancellation of the Tokyo 2020 Summer Olympic Games, which denotes the first time since World War II that the Olympics were postponed [2]. In recent memory, the only time sports in the United States were universally cancelled occurred after the terrorist attacks on September 11th, but even one of the greatest modern American tragedies only paused sports for a matter of days and weeks before they resumed. American sports are currently navigating uncharted territory in the recent postponements of the NBA, MLB, NHL and NCAA, leaving the collective sport industry without athletes due to self-quarantining and being barred from training at team facilities. Beyond the assured financial impact this hiatus imposes on the sports world lies a unique challenge for health and performance staff (e.g., strength and conditioning coaches, nutritionists, and team physicians). While remote training, which the majority of athletes are currently engaged in, is not unique, the notion that virtually all public training facilities are closed is, thereby challenging sport practitioners to progress athletes amidst unfathomable circumstances [5]. This not only hinders the athlete’s strength and conditioning regimens, but also the overall monitoring of other key human performance factors, such as nutrition, sleep, and recovery, that thrive on direct coach-athlete engagement. As such, practitioners are forced to develop new strategies for athlete monitoring that are flexible to the societal impacts of COVID-19 despite a great number of uncertainties [6,7,8,9]. 

Complete return to normalcy post-COVID-19 is only attainable via the development of a vaccine applied globally. Following the initial identification of the virus, current estimates project the arrival of a universal vaccine within the subsequent 12–18 months [10]. However, life and sport are already attempting to resume functioning in a “new normal” before a viable vaccine is introduced. As such, the supposed “new normal” for sport must, at a minimum, establish then meticulously account for the continuous risk of COVID-19 infection throughout the indefinite period of not having access to a vaccine. If carried out properly, these steps will help return sports back to sustainable training and competition for the foreseeable future. Further, many leagues and governing bodies are continuously releasing guidance on policies and procedures for a return to sport as they evolve, which leaves athletes and staff constantly scrambling for strategies to comply [11,12,13]. This is a challenging endeavor for a fully staffed athletics department under normal circumstances, but now must be accomplished during uncertain times with reduced budgets and staff. 

In this commentary, we present a sport science approach to not only complying with guidance as it is released, but remaining fully prepared for the potential of additional alterations to the current status quo. These objectives are accomplished through advanced monitoring tools comprising subjective (e.g., questionnaires) and objective (e.g., force plate assessments and wearable sport technology) monitoring thus enabling the safe return to sport. Specific emphases are directed at the health and safety for the athletes and staff. Perspectives from multiple practitioners and sport scientists regarding data/technology strategies, phases of implementation, and practical examples of data and data visualization are further discussed as well.

## 2. Perspectives

Athlete monitoring strategies that incorporate data analytics and technology provide the greatest opportunity for success when plans are adaptive to the perspectives and overall objectives of the sport practitioners (e.g., Performance Director, Strength and Conditioning Coaches). However, this notion is assuming that there are likely to be moving targets throughout any given calendar year, let alone during these turbulent times. Below are commentaries from two sport practitioners working directly with collegiate (Division-I) and professional athletes, in which they elaborate on their own methods for utilizing data-driven athlete monitoring as a tool for both performance and COVID-19 surveillance.

### 2.1. COVID and Collegiate Strength and Conditioning (S&C): Insights from a Director of S&C at a Power 5 University

Over the last two months, COVID-19 dramatically changed society and how everyone interacts daily, which also carried with it a complete shutdown of the athletic department and normal training regimens for athletes everywhere. As a strength and conditioning coach that is very interactive and uses a hands-on approach with training and coaching athletes, this has been an extremely difficult and challenging time. Normally, the spring to early summer season is especially productive and beneficial for the physical maturation of athletes during a critical phase of their off-season programs. However, with the pandemic, training has become remote and limited for high percentages of the athletes. My approach to training athletes remotely has been to prioritize what are the most important outcomes and the end goal for each athlete. First and foremost, athlete safety and wellness are the most vital and critical components within the daily interactions and programming of workouts and routines. Nutrition and sleep are very important to everyone but to athletes, these components are crucial to staying healthy and maximizing adaptations on a daily basis. Our athletes use sleep tracking and heart rate variability monitoring [14,15], nutrition apps for monitoring and interacting with their daily diets, as well as self-report wellness questionnaires via our Athlete Management System [16] to give daily insights into mental and physical stress states and general well-being. Prescribing workouts has also been challenging considering each athlete’s personal resource limitations (e.g., weight equipment, field access, and regional lock downs). Through the use of an interactive smartphone application [16,17], I am able to prescribe multiple workouts and variations to meet every demand for each athlete ultimately to maximize their resources. The foundations that our athletes use all year further allow me to monitor and help guide each athlete over the very difficult first 8 weeks of the pandemic. All of these tracking tools are fully voluntary for our athletes, but maximizing applied performance has become a fabric of our culture at West Virginia University and our athletes crave the ability to gain an edge in their performance, recovery and daily wellness. Over the next several weeks and months, as society continues to open up and return to a new normal while athletics follows suit, with the importance of return to play (RTP) and preparing athletes to compete again, we will use several technologies to help with athlete performance and safety. We have used external load monitoring via GPS [18] for several years and continue to do so as a guide for player load and proper progressions as athletes transition from injured to full participation. Further, heart rate monitoring [19] will be a major component for us to measure conditioning baselines and athletes’ readiness when they return to campus. This information further aids us in how to prescribe progressions to ensure athletes return to the most physical preparedness safely. It will continue to be a challenging time for athletes to get back to where they need to be. Through the ability to measure internal/external loads, power expression (e.g., routine force plate testing), GPS, lifting percentages and other measurables tied to an athlete’s overall health for eventual comparisons to previous numbers before the pandemic, we will be able to properly assess, monitor, and progress in a safe manner. Our goal and mission for WVU strength and conditioning is to maximize the physical potential of each athlete with an underlying full commitment to athlete safety, therefore making the use of data and technology for performance and monitoring critical.

### 2.2. COVID and NFL Athletes: Insights from a Director of Player Performance in the NFL

As we contemplate how we can safely return players and staff to training facilities, health, wellness, and safety are the priorities. Some logistical concerns for arriving players are their current training status, health, previous exposure to COVID-19, and any unresolved injuries. Consideration must also be given to returning coaches and support staff. Furthermore, current health status, previous exposure to COVID-19, and COVID-19 underlying risk factors must also be addressed. For players and personnel, monitoring will be essential to mitigate risks for any internal outbreak. An additional concern is the player’s current training status. It is customary for players to go through physical assessments prior to training, which is used to establish each athlete’s “new normal”. This is vital, even more so now, since the players had their normal off-season training disrupted and likely experienced some level of deconditioning. More challenges also arise with social distancing and other protective measures being put in place for non-football activities such as weight training.

The implementation of a COVID-19 monitoring program is likely to be standard at most facilities. The use of data, comprising both wearable and non-wearable technologies, will make our process more efficient and effective. Simple daily questionnaires, sleep tracking, heart rate variability, and other vital signs are in place to provide an advantage for detecting an illness early in its onset, ultimately aiding to avoid unnecessary exposure. Incorporating COVID-19-specific assessments into our existing player performance monitoring will also be advantageous for contact tracking and contact tracing should an event occur. There are some logistical hurdles associated with implementing more monitoring, such as compliance, manpower resources, reporting, and facility population (players, coaches, staff, and business staff), not to mention adding to an already hectic schedule. However, the limitations are minuscule compared to the impact the additional monitoring would have, provided athlete safety and wellness are at the forefront of our objectives.

## 3. Approach

One of the most problematic factors in the rapid spread of COVID-19 is the high viral transmission load present in an infected person before symptoms appear [20]. More specifically, reports claim that up to 80% of infected people are completely asymptomatic [21]. An athlete or staff member that feels completely normal conceivably could be COVID-19 positive and asymptomatic (either completely or at the time), while concomitantly exposing numerous athletes and staff members to the virus. By nature, sport involves close physical contact between athletes and staff, whether that occurs in the training facilities or locker room or during plane/bus transport, and of course within the realms of training and competition. Strict procedures such as quarantining an entire team and staff for a whole season are possible, but unlikely for a long duration provided the aforementioned impacts such a hiatus imposes on all of the entities involved in athletics. Even in this best case and improbable scenario, it is impossible to reduce the risk completely. The shutdown of an entire team and/or league is indeed possible if just a single athlete or staff member possesses the SARS-CoV-2 virus and is asymptomatic, as witnessed in the NBA earlier this year.

Here, we present a “digital PPE” sport science-driven approach to monitoring athletes’ physiology, performance, subjective health/wellness, and cognitive state, which culminates into an alert-style dashboard and identifies patterns associated with poor physiological state (e.g., inadequate sleep, elevated night time heart rate, and decreased relative power output), and potentially viral infection. When designed appropriately with athletes’ and practitioners’ needs in mind, self-report measures can be accomplished through minimal burden while providing meaningful and actionable data into athlete health and wellness [22,23]. Additionally, wearable technologies are commonly used in athletics to understand training load and recovery, and are currently under rapid investigation for use in COVID-19 surveillance [24]. In combination, self-report and physiological measures can give specific insight into the athlete’s deviation from homeostasis, which can be linked to under-recovery, overstress, and/or potentially illness. Ultimately, these efforts are devoted towards enacting quarantine procedures on an asymptomatic but infected athlete before they can expose more athletes and staff. The “digital PPE” strategy is not intended to be a medical diagnosis, rather purports use as a quantitative way to prioritize wellness checks for athletes and staff, in which case the organization’s medical procedures should take precedent over this holistic dataset. 

Athlete monitoring, or sometimes referred to as applied sport science, involves a scientifically minded approach to training and competition such that the overall training process is diligently planned, training is quantified and subsequent alterations to the athlete’s preparedness are measured [25]. Detailed athlete monitoring allows coaches to better understand the recovery-stress state of the athlete, as well as various aspects of recovery/adaptation, and illness/injury trends [25,26,27,28]. The ultimate goal of these efforts is to enhance sport performance [29]. Origins of athlete monitoring trace back to several Eastern Bloc countries including Hungary (1940–1950s) and the Old Soviet Union (1950s). More modern examples include the Australian Institute of Sport (AIS), English Institute of Sport (EIS), National Institute of Sport Expertise and Performance (INSEP, France), and Aspire Academy (Qatar). Of note, the AIS is often credited for bringing applied sport science to the forefront of collaborating with academia as observed through their efforts of integrating with the Australian University system. The AIS began after Australia failed to obtain a single medal at the 1976 Olympic Games. In 2000, they won 58 medals at the Summer Games in Sydney. 

The number of full-time applied sport scientists within U.S. collegiate and professional sport has grown rapidly over the last 10 years. Currently, most NBA, NFL, NHL, and MLB teams have a full-time sport scientist(s) and many collegiate athletic programs have either a sport scientist(s), someone(s) with sport science-related tasks built in to their professional responsibilities (e.g., a coach overseeing GPS or a strength-utilizing force plate technology) and/or a collaboration with an academic unit. Additionally, many programs possess some version of a sport performance enhancement group comprising various staff members (e.g., sport medicine, sport coach, strength coach, sport scientist, dietician, and sport psychologist) that each have specifically defined roles. From there, a communication structure is built and aimed at supporting athlete development through an inter-disciplinary and integrated approach. Further improvements in the delivery of sport science are derived from the recent developments in technologies and processes (e.g., smartphone apps for data entry and applied programming interfaces for automated data transfer), which is allowing practitioners to utilize data with less impact to daily schedules. Many tools varying from daily surveys to wearable technologies are currently utilized as part of normal, day-to-day practice. We propose a framework to modify existing athlete monitoring methods to not only continue current practice, but expand capabilities for COVID-19-specific surveillance to enable return to training and competition until a vaccine is available world-wide. Much of this discussion is directed at providing a conceptual framework to combat this difficult situation. Perhaps the most important component is the appreciation of meaningful data collection. Inevitably, every program’s situation will be different to some degree (e.g., finances, expertise, resources, and sport calendar). Indeed, the uncharted nature of the ongoing pandemic provides much uncertainty. Thus, it is our hope that the discussion of a flexible data-driven plan delivers guidance to those already collecting data in a structured manner, enabling them to adjust when/where appropriate, and extends to those under current realizations that more formal data monitoring procedures are warranted. Ultimately, our primary objective is to delineate strategies for athlete monitoring in an actionable fashion, such that adapted efforts are ready for deployment when athletes return. These strategies reported herein are structured merely as recommendations, although they stem from ongoing initiatives designed and employed by the authors.

## 4. Data Strategy

The key areas of the data approach with details are listed in Table 1 below. 

The data sources listed in Table 1 are split into three categories for “purpose”—COVID-19 tracking, performance monitoring, and dual purpose. “COVID-19 Tracking” data sources are specific only to infection detection, “Performance Monitoring” is current standard practice with not as much applicability to COVID-19 (yet remain extremely valuable data), and “Dual Purpose” can be used for both COVID-19 and performance monitoring.

Efficient data aggregation for all of the sources described in Table 1 is a critical component to scaling to a large number of athletes while maintaining the ability to action the data in near-real time. This can be carried out using pen/paper, using free online tools, widely available programs in Microsoft Office (Excel, Word, Powerpoint, Access), up to a central Athlete Management System (AMS). The aggregation method should be selected based on ease of data workflow and availability of funding. An example of a very low-cost solution could be obtaining daily symptom data through the use of free online tools such as Google Docs or Google Sheets, and aggregating the data manually into Microsoft Excel for creation of conditional formatting and graphs for ease of data visualization. A higher-cost solution would be to utilize a commercial AMS, which is designed specifically to aggregate data automatically using smartphone applications, direct entry, and automated data imports using applied programming interfaces (APIs). The advantage to using an AMS is in the ease of workflow for both the athletes and practitioners, where, for example, the athletes fill out a morning symptom (e.g., CDC symptom lists for COVID-19) questionnaire, and if any symptoms are reported, a practitioner could be instantly alerted via text message and/or email as soon as that data is entered. These triggers can be fully customized based on staff needs and philosophies. Examples of several commercial AMSs include Fusion Sport Smartabase (Fusion Sport, Colorado, United States and Brisbane, Australia; utilized and noted in figures below), Kinduct (Kinduct Technologies Incorporated, Halifax, NS, Canada), CoachMePlus (CoachMePlus, New York, NY, USA), The Sports Office (Kitman Labs, Dublin, Ireland), Kitman Labs (Kitman Labs, Dublin, Ireland), and Rock Daisy (RockDaisy, LLC, New York, NY, USA) to name a few.

### 4.1. COVID-19-Specific Tracking

We recommend that current COVID-19 tracking procedures include a combination of daily self-report symptom assessments and periodic biofluid biomarker analysis. For ease of application from an effort and cost of perspective, daily symptom tracking is a critical data point that should be incorporated immediately. The current CDC list of symptoms include the following: cough, shortness of breath or difficulty breathing, fever, chills, repeated shaking with chills, muscle pain, headache, sore throat, loss of sense of taste or smell [30]. This list can be easily distributed as part of a morning wellness questionnaire, which is common procedure in sport, and filled out in less than 20 s. Such data provides vital information regarding new symptoms and/or changes in symptoms that could be indicative of COVID-19. This data can also be immediately actioned by the practitioners via automated alerts if using an AMS platform. As stated earlier, this data should not be used as a diagnostic tool, but as a way to enable wellness checks following the organization’s medical procedures.

Ground truth understanding of contact with the SARS-CoV-2 virus and time course through the body will only come from sampling via nasal swabs and blood. A major component specific to each athletic department/team will be their budget and ability to afford (or not afford) frequent biospecimen testing. Indeed, the financial situation for each program will likely impact their specific approach to monitoring. Nasal swabs will indicate viral load, and blood will determine the concentrations of IgM and IgG, which are indicative of infection (IgM) and recovery (IgG) [31]. This sampling should be performed at regular intervals after the quarantine stage and during return to training/competition such that time points of sampling are consistent throughout to enable for the most effective comparative analyses. 

### 4.2. Performance Tracking

Training and subsequent athlete responses are routinely monitored in sport for both strength and conditioning sessions and on field practices. This is commonly performed via external workload methods, which may include weight room volume load (reps × load), countermovement jumps (force plates) and movement-based technologies (i.e., GPS or RFID), as well as internal workload methods, namely subjective ratings of perceived exertion and/or heart rate monitoring [32,33,34,35]. Additionally, periodic performance-based tests (e.g., force plate monitoring, agility tests, and endurance tests) can be carried out to assess specific adaptive responses, changes in performance and the athlete’s overall preparedness [25,26,36,37,38,39]. Ideally, the training is driven, first by the coach’s periodized plan in which programming strategies are utilized to bring about pre-determined adaptations. Monitoring allows the coach to know (1) what workload was actually completed in training (this can change from the original prescription based on various factors), (2) the individual athlete’s response to the training, and (3) can potentially “head off” unwanted issues before they become catastrophes (a fatigue management issue is identified). Other aspects of athlete monitoring include talent identification, a better understanding of the team as a group (e.g., strength levels of incoming freshmen, and trends in development) and the ability to provide professional evidence. To the last point, recent articles have discussed how athlete monitoring can serve as tool for coach and strength coach evaluation [40,41,42]. 

While these monitoring strategies are well documented for use in athletics, training load tracking becomes increasingly important during the COVID-19 return to training and competition phases. The quarantine phase elicited potential down time with athletes, who at best are training on their own and likely with limited resources (e.g., no access to free weights). The adapted and trained state of the athletes are affected and should, therefore, be monitored closely as athletes return to training. Many athletes are relegated to body weight exercises and for many sports actual sport practice is impossible. Therefore, objective and subjective monitoring and frequent communication during the quarantine period likely helps the athlete remain “connected” and provide the coach with a better idea of what the athlete’s training looks like. It is important that this monitoring and communication comes from a very supportive place and that the athlete does not (1) feel that the monitoring is overbearing or intrusive, and (2) that athlete should understand that their health is paramount over training and should not feel guilty if their training and overall performance are hindered. 

Consequences of detraining have been well investigated in the literature (Fleck, 1994, Madsen et al., 1985, McMaster et al., 2013; Mujika and Padilla, 2000a,b). The ability to capture potential decrements in adaptation(s) and performance upon return will require athlete assessment and performance measurement. Ideally, (most of these) tests (which may need to be modified early on) were performed prior to quarantine allowing for post-quarantine comparisons to be made. A detailed overview of the many relevant tests that coaches may use to assess their athletes is beyond the scope of this article. In the section “Phase III: Return to strength and conditioning” (below) we attempt to describe a few real-world aspects of athlete assessment strategies through the lens of returning to strength and conditioning training. For a detailed look into assessing maximal and ballistic strength (McMaster et al., 2014), sprinting (Haugen and Buccheit, 2016) and conditioning (Buccheit, 2008), there are indeed many helpful resources for practitioners. Additionally, we strongly encourage coaches to refer to the National Strength and Conditioning (NSCA) and Collegiate Strength and Conditioning Coaches Association (CSCCa) joint taskforce paper on “Consensus Guidelines for Transition Periods: Safe Return to Training Following Inactivity [43].” Of note is (1) the importance of baseline assessment and (2) that traditional strenuous conditioning tests should be modified allowing for reduced work and stress (NSCA). Similarly, upon arrival, measurements should be made in the weight room but one repetition maximum (1RM) testing should be avoided. Measurements on the field and in the weight room allow for performance tracking to be individualized. Periodically reassessing over time allows for training to be adjusted appropriately based on evidence gained through the data collection process. Not only can detraining rates differ for various athletes and for various adaptations but gains accrued through the first several weeks and months may also differ due to the athlete’s physiology, training history and approach to training.

### 4.3. Dual-Purpose Tracking

There is a clear intersection between performance monitoring and COVID-19 tracking in sport, and that resides in health/wellness, sleep/sleep physiology, and cognitive testing data, although it is likely that maladaptive indices within any of these domains will likely influence exercise and sport performance [29,44,45]. Each has an application in sport for performance monitoring, while also serving as tools that can be indicative of deviation from homeostasis, or potentially illness. Health/wellness tracking typically consists of a daily survey that is performed verbally, using pen/paper, or ideally smartphone app for ease of centralized data organization. Questions on a Likert scale are commonly applied in performance domains and give an indication of key metrics such as soreness, fatigue, motivation, and sleep [22]. These metrics can be slightly refined to have dual purpose with COVID-19 symptom tracking, where fatigue and body pains are associated symptoms. Therefore, modifying and adding an additional set of symptom questions to a morning wellness questionnaire is a simple and effective strategy to account for both performance and COVID-19 monitoring. Similarly, quantified sleep tracking is more common in performance monitoring, and is available through many different commercially available devices. Sleep is an important factor in recovery such that it should be tracked either subjectively (wellness questionnaire), objectively (wearable device), or ideally both. 

What is more important for dual-purpose application is tracking sleep physiology metrics, which include but are not limited to heart rate (HR), heart rate variability (HRV), respiration rate (RR), and temperature. During illness, all of these metrics will be significantly altered, with an elevated HR, depressed HRV, elevated RR, and, of course, a fever [46,47,48,49]. Methods for understanding deviations in these values are addressed in the upcoming Implementation section. For performance monitoring, HR and HRV are associated with factors of recovery from training loads as well, which also magnifies the utility of measuring training load. For these reasons, sleep physiology is essential for dual-purpose tracking. Finally, cognitive testing apps are applied to give indications of mental status via working memory and reaction time, both of which are correlated to performance and illness [44,50]. 

## 5. Phases

Sport in the age of COVID-19 can be broken down into multiple phases, each of which has distinct differences that need to be carefully monitored, which can be performed using a combination of the datasets previously listed in Table 1. 

### 5.1. Phase I: Quarantine

The quarantine phase is what the sport world was instantly introduced to upon the cancelation of the NBA season, with the NCAA, NHL, and MLB soon to follow. Competition was canceled while, in most cases, facilities closed down to training and practice, only remaining open for high priority injury rehabilitation. Athletes are no longer able to be trained by strength and conditioning coaches in the weight room, cannot receive non-injury treatments from trainers, no longer have access to high quality nutrition provided in the facilities, and miss the camaraderie from teammates and staff. All of these are factors that relate to not only physical but mental stress thus need to be monitored.

### 5.2. Phase II: Return to Facility

The second phase begins when the decision is made to re-open facilities to athlete access. Considerations need to be made to understand if the athletes and staff have been previously exposed to COVID-19, which can be achieved through blood-based antibody testing [31]. Current methodologies include a venous blood draw and central lab analysis or a more rapid lateral flow assay (LFA). Due to the rapid nature of the development of these diagnostics, any recommendations should be guided by the local medical staff of the organization. Since this phase is the first interaction of the athletes with staff, controls should be provided to limit the exposure of athletes to each other, which can be achieved through the logistics of scheduling medical screens. However, this is the phase where it is critical to closely monitor COVID-19 symptoms via the Daily Wellness Questionnaire and physiology linked to COVID-19 via wearable technologies. Acute changes in either of these datasets need to be immediately available to the staff for a more detailed wellness check where organization medical policies take over. This strategy should be continued throughout the remaining phases. 

### 5.3. Phase III: Return to Strength and Conditioning

After medical screening and return to the facility, on-site strength and conditioning in preparation for the start or resumption of practice and competition begins. In addition to all of the data in Phase II, screening of IgG and IgM antibodies should be tested at regular intervals for any athletes and staff that did not test positive for IgG in the first screening. This enables continuous tracking of athletes that may have been exposed and would have the potential to spread to the rest of the group. This is the first phase, where controlled interaction between athletes is likely to begin, so monitoring any potential exposure to COVID-19 becomes critical. Continued monitoring of subjective symptoms and sleep physiology should also be used as indicators for when additional medical wellness checks can be administered. Sudden changes in symptoms, and/or changes in resting heart rate, heart rate variability, temperature, and respiration can be potential indicators of illness. These tools should not be used directly as a diagnosis, but should be used to alert the medical staff to perform a check.

Baseline performance screening to assess an athlete’s initial conditioning and neuromuscular characteristics provides baseline data to provide coaches insights on their athletes’ training status. The battery of tests should include force plate assessment to ascertain strength and power capabilities as well as fatigue resiliency under load [26,27,37,51,52,53]. Ideally, the battery of testing is performed periodically throughout the year so that when the athletes return coaches are able to compare current performance data to previous data collected before the athletes left for quarantine. It is important to note highly talented and developed athletes very well may perform impressively but be in a poorly trained physiological state [54]. Thus, the change in performance is critical, perhaps more so than simply what the value(s) for a given test may be. Initial strength assessments for incoming college freshmen athletes have been discussed in the literature in large part due to injury rates being higher for collegiate freshmen, particularly for fall sports in which the competition phase often begins right when the athlete arrives to campus. Stone et al. [55] discussed the benefits to assessing maximal strength via an isometric clean grip mid-thigh pull because it allows for the assessment of peak force (maximal strength) without having the athlete perform a 1 repetition maximum (RM). Indeed, 1 RM testing, immediately when the athletes return, is likely unsafe due to the likelihood that many athletes have been unable to perform resistance exercise with heavy loads. Kraska et al. [51] demonstrated that when athletes perform a series of vertical jumps across a spectrum of loads that weak athletes, even slightly loaded (20 kg, an empty barbell), “drop off” a substantial amount (e.g., peak power output drops >30% between from unloaded and loaded 20 kg condition). Indeed, weaker athletes are at greater risk of experiencing non-contact injuries due to the inability to withstand eccentric braking forces during high velocity change of direction movement tasks [28,52,56,57,58]. This risk is increased when the athlete is heavily fatigued [59,60,61]. 

Monitoring training load during Phase III is essential for understanding the physical demands imposed on the athlete during training. With limited control of training leading into this phase, and the potential for compressed practice schedules, training data should be monitored and used as microcycle and mesocycle guides to understand each athlete’s progression (or lack thereof) to determine when they are ready for higher workloads, or potentially lower. Ideally, there is ample time for athlete physical development prior to beginning sport-specific practices such that strength and conditioning regimens are used to better prepare the athletes for the demands of the sport [62,63]. For both returning to team strength and conditioning and returning to practice training, physical demands should begin at conservative levels and gradually increase over the first several weeks [63]. The National Strength and Conditioning Association noted that training and conditioning mistakes (e.g., injury or death due to overtraining) commonly occur when returning from extended breaks (e.g., summer and winter) and during coaching transitions [41,43]. Additionally, previous research noted that some of the more severe medical-related training catastrophes occur when a newly hired coach arrives with an ambition to “send a message” early on [41,43]. 

### 5.4. Phase IV: Return to Practice

In some cases, it is certainly possible that Phase III and IV occur in tandem depending on the sport. Still, when team practices resume, this presents an additional level of risk for athletes due to the largely unavoidable interaction between athletes. Following initial implementation, all of the COVID-19 monitoring tools from the previous phases should continue to help limit the risk of exposure. From a training perspective, additional workload monitoring tools such as GPS devices and force plate testing (external) and/or subjective ratings and heart rate monitors (internal) should be used if available to continue the workload tracking during conditioning sessions and field practice. Additionally, heart rate monitoring technology can be used to more closely monitor any potential heat injury issues during practice [64,65,66,67].

### 5.5. Phase V: Return to Competition

Finally, the highest risk to both athletes and staff for COVID-19 infection is when competition resumes, which inevitably will require both travel and close interaction with a completely new group of athletes from another geographic location. Ideally, a comprehensive monitoring strategy will be enacted league wide to reduce overall risk from the interaction between at least two different teams. Table 2 below describes the Phases of COVID-19 in sport.

## 6. Implementation of COVID-19 Sport Science Strategies

**Figure 1:**
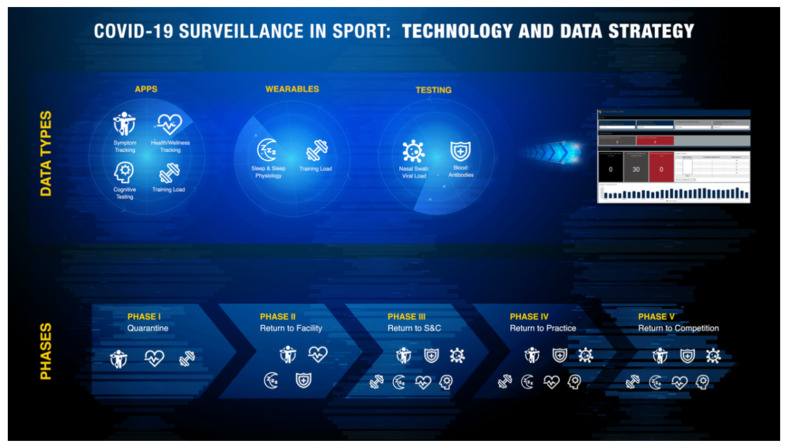
COVID-19 Data Monitoring Strategy.

Implementation of COVID-19 monitoring in conjunction with the aforementioned performance monitoring strategies consists of two main steps, measure and track. In this section, we will provide an example of specific ways to track symptoms, health/wellness, and sleep/sleep physiology. Technology based training load tools are assumed to be standard practice and handled by current S&C staff, and blood/nasal swab testing should be designed and implemented by the medical staff. The various components that comprise general strategies to effectively execute holistic data monitoring throughout Phases I–V are depicted in Figure 1. 

### 6.1. Measure: COVID-19 Symptom Tracking

**Figure 2:**
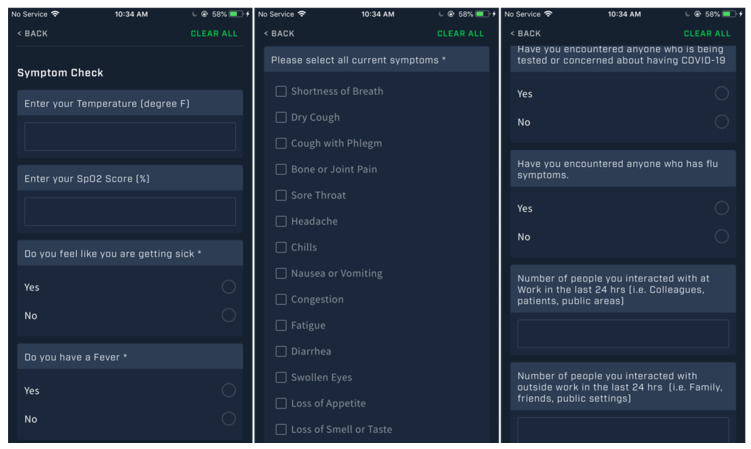
COVID-19 Monitoring and Symptom Daily Questions.

It is important to retain flexibility in symptom questionnaires as the CDC is likely to provide updated symptom lists over time [30]. In addition to symptom questions, additional information such as oral temperature, blood oxygenation via pulse oximeter, and any interactions with others that are suspected as possibly being ill are important data points. Based on current guidelines, the following set of questions shown in Figure 2 (utilizing Fusion Sport Smartabase) is suggested as an example that can be implemented in a Daily Wellness Questionnaire:

### 6.2. Measure: Health and Wellness

**Figure 3:**
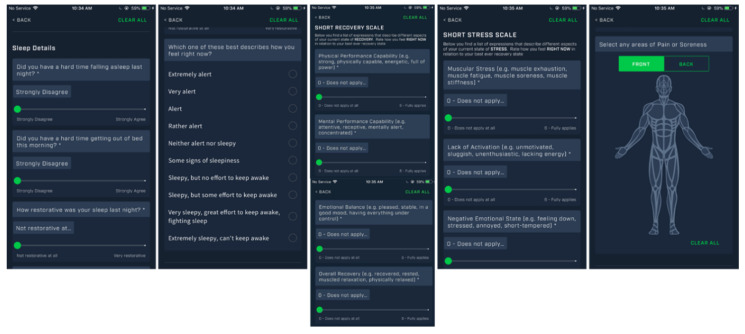
Athlete Health/Wellness Daily Questions.

Dual-use purpose of understanding the overall health and wellness of the athlete is important for both performance and COVID-19 monitoring. This can be achieved by understanding subjective sleep restoration, daily energy levels, and any soreness or pain experienced by athletes via interactive anatomical body diagrams. For understanding the recovery/stress state, the Short Recovery Stress Scale (SRSS) can be used [68]. This is a strongly validated tool that gives insight into Physical, Mental, Emotional state in less than 30 s of the athlete’s time. Figure 3 below (utilizing Fusion Sport Smartabase) provides an example of dual-use health/wellness questions as part of a Daily Wellness Questionnaire.

### 6.3. Measure: Sleep Behaviors and Sleep Physiology

**Figure 4:**
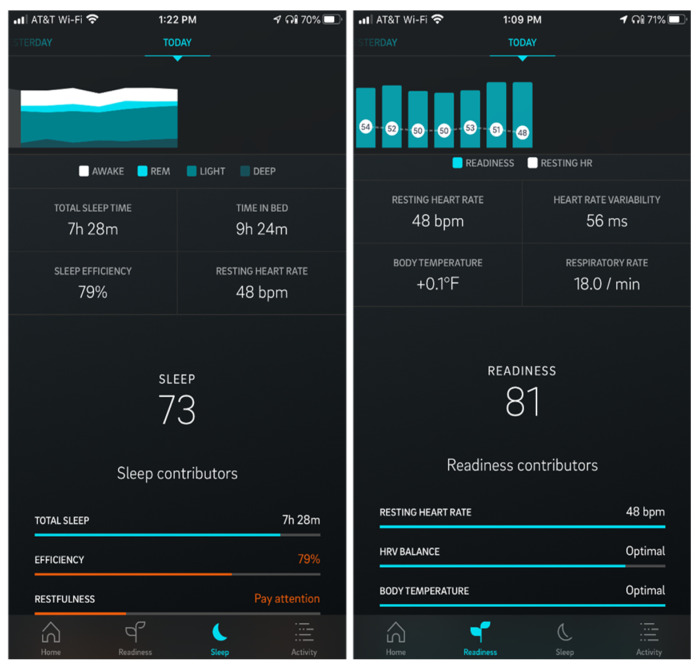
Sleep/Sleep Physiology Data.

Sleep is certainly one of, if not the most, important factors in recovery. For athletes and staff, it is important to consider that sleep can be characterized by quantity (e.g., time spent in bed and total sleep duration) and quality (e.g., heart rate (HR), heart rate variability (HRV), respiratory rate (RR), and temperature). Sleep physiology, therefore, allows for direct dual use between performance and COVID-19 monitoring. Important metrics that can be tied to performance and illness include the aforementioned sleep physiology variables HR, HRV, RR, and temperature. An example device that includes these metrics is the Oura smart ring [14], which is currently being investigated in COVID-19 studies [69,70]. Figure 4 below illustrates an example of sleep/sleep physiology data via smartphone app.

### 6.4. Track: Data Approaches

Once data is obtained as described in the Measure section above, rapid data visualization and simple analytics approaches can be utilized for quick actioning. There are two main types of alerts that can be applied for this holistic dataset: discreet and intra-individual changes. Discreet changes apply to a small but critical number of variables that represent a “Yes” or “No” response. These include self-report questions: Do you feel ill? Do you have a fever? Have you encountered anyone who has flu symptoms or is being treated for COVID-19? In the event of “Yes” responses, an immediate alert should be sent to the staff member(s) tasked with health/wellness tracking of the athlete. COVID-19 symptoms should also be tracked as discreet changes, where the event of a new symptom is important information for the staff to immediately review. Due to the medical nature of this question, the parameters for alerts in tracking symptoms should be designed by the medical staff. The second approach to data calculation and visualization is pertinent for parameters that are unique and variable to the athlete, such as intra-individual physiological metrics obtained by wearable devices. For example, a HRV measure should not be compared as an absolute value between athletes, but needs to be tracked as intra-individual fluctuations. Sands et al. [26] present vast evidence for methods of data tracking of athletes supporting the need for establishing both intra-individual and meaningful worthwhile changes in the context of a singular elite athlete, which is then replicated for all athletes on any given team sport. For physiological parameters, one approach includes examining today’s (e.g., most recent record) data compared to the rolling average for that athlete to understand the change relative to that athlete’s historical normative values. One simple method for this comprises calculating a Z score for each meaningful parameter, which is the current value minus the average, divided by the standard deviation. A score of 1.0 means that today’s value is one standard deviation above the athlete’s average, whereas a score of −1.0 suggests that the most recent score is one standard deviation below the historical average. It is important to consider the context of the Z score, as there are instances in which a score that is higher or lower than the average may be desired depending on the situation at hand. For example, a Z score of −1.0 following a timed 40 yard sprint would suggest that the speed of the athlete drastically improved (e.g., time of the sprint decreased) relative to all of the past performances yet a Z score of −1.0 with respect to sleep duration infers the athlete obtained considerably less sleep the night in comparison to their normal sleep hygiene. As such, a Z score of −1.0 could be a positive or negative indication (same applies to 1.0), and thus practitioners must understand the context of each score. The challenge for the current COVID-19 pandemic lies with the real-time understanding of the effects of the virus on humans, let alone athletes. Because of this, it is currently not achievable to have a standard value of change (e.g., Z score) that is indicative of COVID-19. However, the nature of conditional formatting with Z scores allows for the staff to assess the “alert” value of change with parameters linked to illness, such as temperature, resting heart rate, heart rate variability, and respiration rate. The intent of “alert” values is for staff to initiate triaged wellness checks, so it is likely that false positives will occur at a high rate. These can eventually be balanced out once more is understood about COVID-19 and further data disseminating from the aforementioned sources are collected on individual athletes.

### 6.5. Track: COVID-19 Symptoms

**Figure 5:**
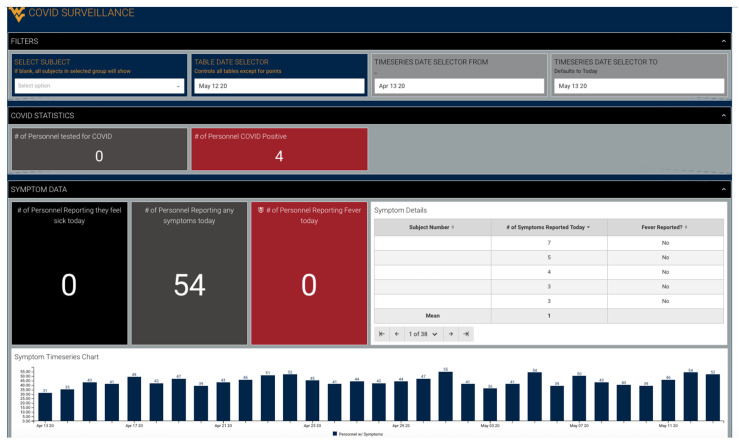
COVID-19 Symptom-Tracking Dashboard.

For COVID-19 monitoring, it is critical to understand any changes in symptoms as they are entered in real time. This can be achieved easily using an Athlete Management System (AMS), as shown in the example dashboard below. Some responses should be treated as instant alerts, such as self-reporting that they feel ill or have a fever. For COVID-19 symptoms, it is important to understand how many symptoms they are reporting, and any sudden changes in symptoms. While some symptoms alone, such as headache, may not be solely indicative of illness, a combination of multiple symptoms and changes from day to day are important to investigate. For the instant alerts, these should also be used as real-time alerts through sending automated emails and/or text messages from the AMS. For example, if an athlete reports a fever, an instant notification should be sent to the staff. Figure 5 below shows an example dashboard (showing manufactured data utilizing Fusion Sport Smartabase for illustration) for practitioners to view team COVID-19 symptoms. 

### 6.6. Track: Sleep Behavior and Sleep Physiology

**Figure 6:**
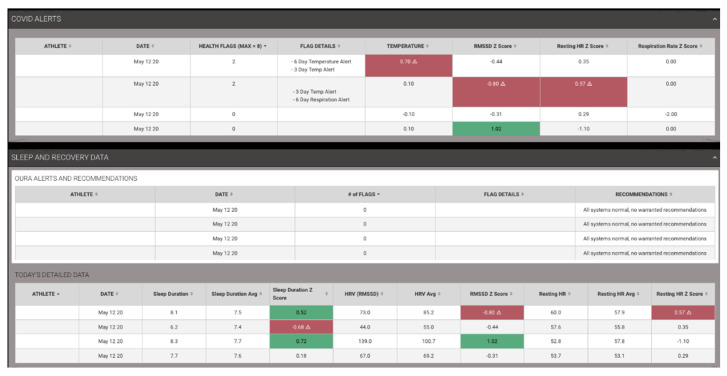
Sleep Physiology COVID-19 Example Alert Dashboard.

Data about sleep that can be objectively measured using sleep wearables including quantity, quality, and physiological metrics such as heart rate, heart rate variability, temperature, and respiration can give indication of stress, under-recovery, and/or potentially illness. As discussed previously, this information should not be used to directly diagnose illness, but can be used for triaging which athletes require a wellness check. While some guidelines may be provided about adequate sleep durations based on the plethora of previous research demonstrating the fundamental importance of sleep for athletic performance [45,71,72,73,74,75,76,77,78,79,80], for purposes of COVID-19 tracking, it is the changes in the sleep and sleep physiology data specific to that athlete that is most important to look at for health/wellness/illness monitoring. By utilizing meaningful worthwhile changes and Z scores described above, simple conditional formatting tools can be applied to help guide the visuals, as shown in Figure 6 below (utilizing Fusion Sport Smartabase). Additionally, these deviations can be summed up to provide an overall “Flag” score. For the example below (displaying manufactured data for illustration), we are looking at changes in physiology that are happening multiple days in a row. If the athlete’s temperature has been elevated for 3 days in a row, heart rate variability has been low for 3 days in a row, etc., the combination of these occurrences cause alerts to be identified. For each one of these alerts, a flag is calculated and summed, allowing for auto-sorting to help quickly identify which athletes should be checked on. 

**Figure 7:**
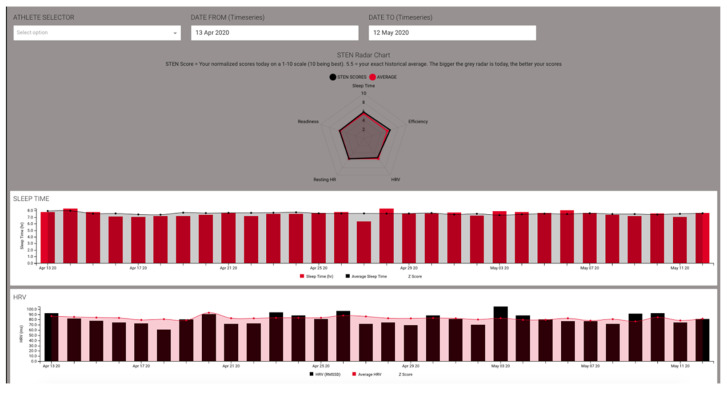
Sleep and Sleep Physiology Time-Series Dashboard.

Additional investigation into an athlete’s data can be visualized on a time-series graph, as shown in Figure 7 below. This allows visual interpretation of changes over time.

## 7. Conclusions

This challenging time of a global pandemic and the inevitable return to sport has created a need to develop new standard operating procedures to take care of athletes and staff members. Guidance from governing bodies and organizations will continue to be released as new data is available, requiring staff to adapt quickly. Applying concepts from the sport science community, and adapting to include COVID-19-specific monitoring parameters can allow staff to not only comply with guidance, but to provide an additional level of protection to the athletes through detailed and continuous monitoring. As additional data becomes available with predictive algorithms based off of wearable devices, new testing tools come online, a good sport science approach as described can immediately implement these solutions and stay adaptive to new science and guidance. However, these tools alone, while powerful, do not replace good practice and expert investigation. The reliability of the aggregated data is only as good as each of the components, and requires careful education of both the staff and athletes for proper data input and assessment. The athletes must be informed of the importance of the data, and the reason for collecting it, and the same applies to the staff [81]. There will no doubt be false positives and false negatives from the data, but it is important that the data be used only to initiate wellness checks and not be used as an absolute diagnosis. That can only be achieved with medical practitioners and approved tests. 

## Figures and Tables

**Table 1:** Data Types and Details.

Data Type	Purpose	Form Factor	Frequency of Data	Description of Data
Symptom Tracking	COVID-19 Tracking	Smartphone App, Spreadsheet, Pen/Paper	1–2× daily	Subjective symptoms following CDC guidelines Currently 18 Yes/No questions
Viral Load Testing	COVID-19 Tracking	Nasal Swab	Every 2 weeks, or onset of symptoms	Quantitative assessment of COVID-19 diagnosis
Antibody Testing	COVID-19 Tracking	Blood (venous or finger prick)	Every 2 weeks	Quantitative assessment of IgM and IgG antibody time course
Health/Wellness Tracking	Dual Purpose	Smartphone App, Spreadsheet, Pen/Paper	1–2× daily	Subjective stress and recovery assessments Generally, 3–10 questions using Likert scales
Sleep and Sleep Physiology	Dual Purpose	Smart Ring	Pervasive during sleep	Objective measures of sleep quantity, quality, heart rate, heart rate variability, respiration rate, and temperature
Cognitive Testing	Dual Purpose	Smartphone App	1–2× daily	Objective measures of reaction time, working memory, and impulsivity
Training Load	Performance Monitoring	Wearable Technology, Smartphone App, Spreadsheet, Pen/Paper	Per training session, weekly to monthly for performance assessments	External (motion, force plates) and internal (heart rate) workload measures during training

**Table 2:** Phases of COVID-19 in Return to Sport.

Phase	Challenge	Main Objectives	Data Acquired
I: Quarantine	No access to facilities, staff	Deploy and monitor training plans Monitor physical and mental health and wellness	Training Load Daily Wellness + COVID-19 Symptoms
II: Return to facility	Limited access to facilities, first concern for exposure to COVID-19	Screen athletes for previous exposure Assess physical condition to begin planning to return to training	COVID-19 Antibodies Daily Wellness + COVID-19 Symptoms Medical Screening Sleep/Sleep Physiology
III: Return to S&C training	Limited control of chronic training workload during Phase I Moderate level of interaction between athletes and staff raising risk of virus spread	Ramp athletes back into S&C safely Keep risk of virus spread low	Training Load Wellness COVID-19 Symptoms Sleep/Sleep Physiology Cognitive Testing Bi-Weekly Antibody Testing
IV: Return to practice	Managing training loads in potentially compressed timeframe in preparation for competition High level of interaction between athletes	Keep athletes safe from acute heat and musculoskeletal injury during practice Keep risk of virus spread low	Training Load Wellness COVID-19 Symptoms Sleep/Sleep Physiology Cognitive Testing Bi-Weekly Antibody Testing
V: Return to competition	Highest risk factor for exposure to virus due to travel, interacting with athletes from other teams/cities	Optimize player performance Reduce risk of virus exposure	Training Load Wellness COVID-19 Symptoms Sleep/Sleep Physiology Cognitive Testing Bi-Weekly Antibody Testing
